# Complete recovery after glucocorticoid replacement therapy in a case of primary adrenal insufficiency caused by adrenal tuberculosis infection

**DOI:** 10.1530/EDM-23-0112

**Published:** 2023-12-13

**Authors:** Hendra Zufry, Putri Oktaviani Zulfa, Rosdiana Rosdiana, Krishna Wardhana Sucipto, Agustia Sukri Ekadamayanti, Sarah Firdausa

**Affiliations:** 1 Division of Endocrinology, Metabolism, and Diabetes, Department of Internal Medicine, School of Medicine/Dr. Zainoel Abidin Hospital, Universitas Syiah Kuala, Banda Aceh, Indonesia; 2 Innovation and Research Center of Endocrinology, School of Medicine, Universitas Syiah Kuala, Banda Aceh, Indonesia; 3 Department of Internal Medicine, Tengku Abdullah Syafii Hospital, Beureunuen, Pidie, Aceh, Indonesia

**Keywords:** Adult, Male, Asian - other, Indonesia, Adrenal, Adrenal, Unusual effects of medical treatment, December, 2023

## Abstract

**Summary:**

Symptoms of primary adrenal insufficiency (PAI) are commonly nonspecific, causing the disease to be misdiagnosed or often delayed, and patients may present to the hospital with a life-threatening crisis. Previous case reports have documented that patients in this condition often require lifelong glucocorticoid replacement therapy. This study aimed to present a noteworthy outcome of PAI caused by adrenal tuberculosis infection, demonstrating complete recovery after six months of glucocorticoid replacement therapy. A 38-year-old Indonesian man presented to the endocrinology clinic in a tertiary hospital with a chief complaint of epigastric pain. The patient experienced nausea, vomiting, loss of consciousness, weight loss, excessive sweat, decreased appetite, weakness, and dizziness in the past 2 weeks. Laboratory examinations revealed hyponatremia, elevated adrenocorticotropic hormone, and suppressed morning plasma cortisol level. A non-contrast-enhanced abdominal MRI showed unilateral right-side adrenal enlargement and calcification. The patient’s Mantoux test was positive. Corticosteroids and anti-tuberculosis therapy were administered. After 6 months, hydrocortisone was discontinued due to the patient’s good clinical condition and normal morning plasma cortisol levels. After a 1-year follow-up, the patient remained asymptomatic with normal cortisol levels. We hypothesized several reasons for this unique outcome: (i) the patient was relatively young compared to previous cases, suggesting an adequate immune system may play a role; (ii) despite a 1-month delay in diagnosis and treatment, the absence of skin hyperpigmentation suggested an acute presentation, potentially contributing to the favorable outcome; and (iii) the absence of comorbidities potentially positively impacted the patient's outcome.

**Learning points:**

- Symptoms of adrenal insufficiency are often nonspecific and may only become apparent once significant damage has occurred to the adrenal gland.
- Clinical adjustments and a comprehensive understanding of epidemiological knowledge are necessary for diagnosing patients with endocrine diseases in limited-resource settings.
- Complete recovery in primary adrenal insufficiency caused by tuberculosis infection might be due to younger age, acute presentation, and absence of comorbidities

## Background

Primary adrenal insufficiency (PAI) is a rare disease that can potentially cause life-threatening conditions (1). Adrenal insufficiency occurs when the adrenal cortex fails to produce enough cortisol due to gland dysfunction or damage (i.e. PAI) (2). PAI is often misdiagnosed due to its clinical manifestations being similar to other diseases (3). Despite global efforts to combat tuberculosis (TB), the disease continues to pose a considerable health burden, with around 10 million new cases reported each year (4). The prevalence of PAI is estimated at 82–144 cases per million, with TB being the leading cause in developing countries (5, 6).

To the best of our knowledge, recent studies have only reported ten PAI cases caused by adrenal tuberculosis infection. Symptoms of adrenal insufficiency are often nonspecific and may only become apparent once significant damage has occurred to the adrenal gland (at least 90%) (5). Unfortunately, this implies that diagnosis is often delayed, and patients may present with life-threatening conditions. Early detection of PAI is therefore critical for better outcomes.

Previous case reports have documented the patient in this condition often require lifelong glucocorticoid replacement therapy. The case report (CARE) Guidelines are followed in this case report, presenting a patient with PAI caused by adrenal tuberculosis infection. Remarkably, the patient achieved complete recovery, and glucocorticoid replacement therapy was discontinued after 6 months, marking a noteworthy outcome. Additionally, a review of the diagnostic test for this specific condition is also discussed.

## Case presentation

A 38-year-old Indonesian male patient was presented to the endocrinology clinic at a tertiary teaching hospital in Banda Aceh, Indonesia, with a chief complaint of epigastric pain. The patient experienced nausea, vomiting, loss of consciousness, weight loss, excessive sweat, decreased appetite, weakness, and dizziness in the past two weeks. Skin hyperpigmentation was not found in the patient. Despite being referred to two secondary hospitals, a definitive diagnosis was unable to be made due to the unclear clinical manifestations. Polyuria was presented, occurring 5–10 times a day and occasionally reaching volumes as high as 4500 mL per day. Anxiety persisted in the patient for over a decade with the fear of driving and heights. There were no comorbidities identified and no history of active tuberculosis (TB) pulmonary infection was documented. The patient’s family members have no similar condition as the patient. Physical examinations were within normal limits.

## Investigation

**Figure 1:**
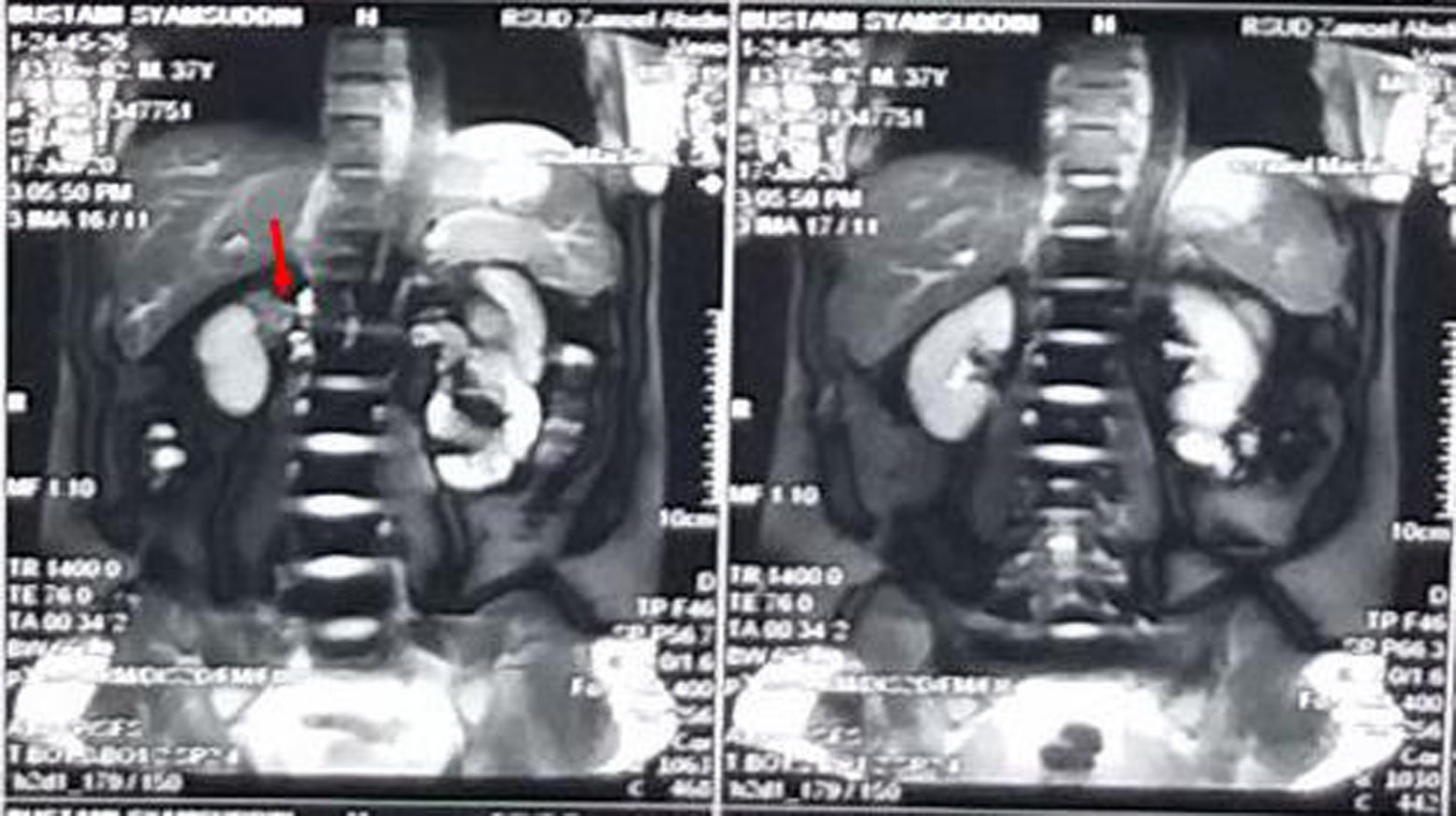
Abdominal MRI examination showed right-side adrenal enlargement and calcification (red arrow).

Laboratory examinations showed results of hyponatremia (107 mmol/L, reference range (RR): 136–145 mmol/L), elevated adrenocorticotropic hormone (785 pg/mL, RR: 0–46 pg/mL), and suppressed morning plasma cortisol (1.2 µg/dL, RR: 3.7–19.4 µg/dL). A non-contrast-enhanced abdominal MRI revealed unilateral right-side adrenal hypertrophy and calcification (Fig. 1). The patient’s Mantoux test results were positive. Chest CT scan and x-ray results showed no pulmonary abnormalities. Based on clinical investigation, the patient was diagnosed with PAI caused by adrenal TB.

## Treatment

**Figure 2:**
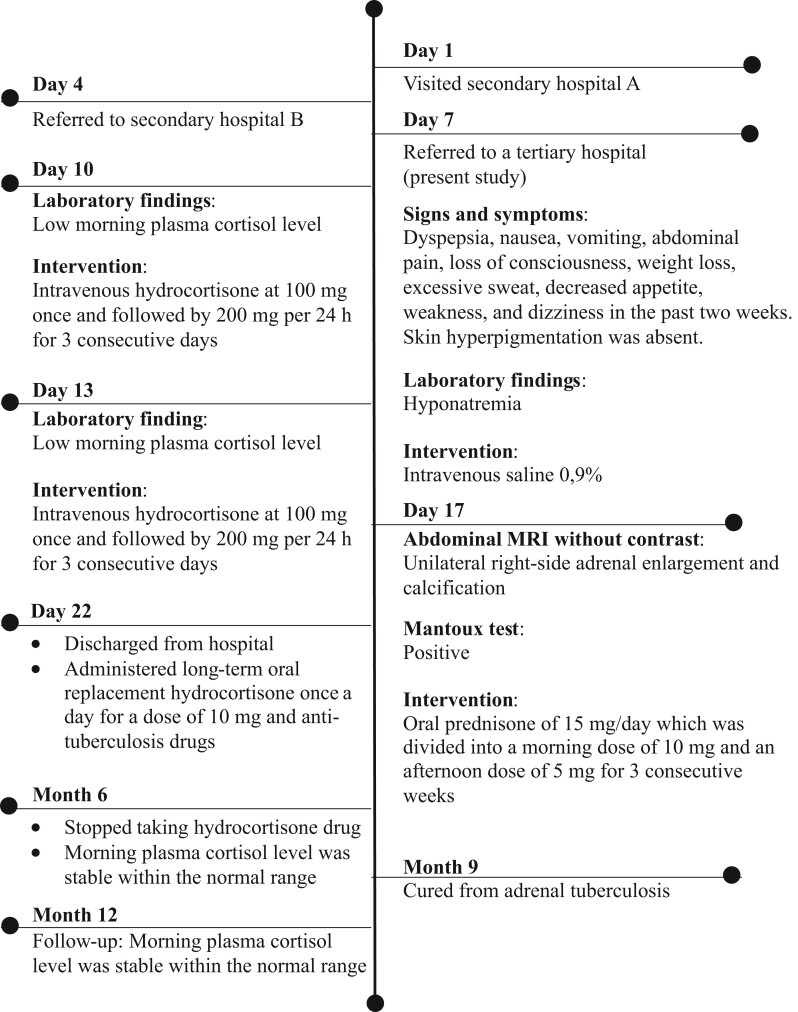
Timeline of diagnosis, intervention, and outcome of the patient.

Intravenous saline 0.9% was immediately administered to ameliorate hyponatremia. The patient received a single intravenous hydrocortisone dosage of 100 mg, followed by 200 mg every 24 h for 3 days. For 3 weeks, the patient was switched to 15 mg/day of oral prednisone; the dose was divided into a morning dose of 10 mg and an afternoon dose of 5 mg. After 15 days, the patient’s morning plasma cortisol level increased from 1.2 g/dL to 10.5 g/dL and was discharged from the hospital. The patient was given 10 mg of long-term oral replacement hydrocortisone once a day. The patient was also administered anti-TB medications (rifampicin, isoniazid, pyrazinamide, and ethambutol) for 9 months. To clarify, Fig. 2 shows the timeline of diagnosis, intervention, and outcome of the patient.

## Outcome and follow-up

The attending endocrinologist (HZ) maintained regular phone follow-ups with the patient; the patient reported excellent adherence to corticosteroids and anti-TB medication. The patient reported no recurrence of previous symptoms six months after initiating hydrocortisone, and morning plasma cortisol levels were within the normal limits (RR: 7.2–63.3 pg/mL); hence, the consumption of hydrocortisone was discontinued. Furthermore, after receiving anti-TB treatment for 9 months, the patient was documented free of TB. The patient had no adverse effects as a result of the treatment. On the 1-year follow-up, the patient remained asymptomatic, and the morning plasma cortisol level was consistently within the normal range.

## Discussion

PAI is an uncommon condition, and its clinical signs may be unfamiliar to certain healthcare practitioners. The case presented in this study was challenging due to the patient’s nonspecific symptoms of PAI, causing the patient to be referred to two secondary hospitals without a definitive diagnosis. The clinical symptoms of PAI are gradually progressing, such as fatigue, weakness, loss of appetite, weight loss, nausea, vomiting, and postural hypotension; consequently, PAI can be easily misdiagnosed as other disorders with similar symptoms (7). Skin hyperpigmentation is present in up to 94% of cases of PAI and can be used to differentiate it from secondary adrenal insufficiency (8). Gastrointestinal symptoms were the primary concern for the patient in this case, and the absence of skin hyperpigmentation made diagnosis challenging. The presence of hyponatremia prompted the attending endocrinologist (HZ) to order cortisol level and adrenocorticotropic hormone testing, which ultimately led to the diagnosis.

To diagnose a case of PAI, in addition to hormonal level tests, the primary test that should be performed is the 21-hydroxylase (21-OH) autoantibodies test. This test helps determine whether the disease is caused by autoimmune disease, malignancy, TB, and so on. However, the 21-OH antibody test is rarely available in limited-resource settings, which were not performed for the present patient. However, we were able to diagnose the patient with PAI caused by TB without conducting the 21-OH autoantibodies test. Indonesia has been listed as having the second largest TB burden globally. Moreover, most cases of autoimmune Addison’s disease tend to affect young and middle-aged women, but this case presented in a 38-year-old male patient. Therefore, clinical adjustments and a comprehensive understanding of epidemiological knowledge are necessary for diagnosing patients with endocrine diseases in limited-resource settings.

The patient was able to be diagnosed using simple diagnostic modalities. In real-world practice, especially in developing countries, access to more advanced diagnostic tests, such as corticotropin stimulation tests, TB tests, CT scans, and MRI, may be limited due to financial and resource constraints. As a result, the lack of accessibility to advanced diagnostic tests may lead to delayed diagnoses of PAI in primary or secondary health-care facilities. The Mantoux test is a low-cost and easily performed option that is sensitive but not specific for the diagnosis of active TB (20). By considering the patient’s clinical presentation, morning plasma cortisol levels, Mantoux test results, and MRI imaging result, the attending endocrinologists (HZ, KWS, and AED) had sufficient evidence to diagnose PAI caused by TB infection.

A pathological biopsy is often necessary to establish a definitive diagnosis of adrenal insufficiency (21). Histopathological and immunohistochemical analysis revealed primary adrenal TB; but the procedure was not performed in this case due to financial and facility constraints. Measuring adrenocorticotropic hormone levels is the first step in diagnosing adrenal insufficiency, and in PAI, these levels are typically above 100 pg/mL (22 pmol/L) (2, 22). Additionally, elevated plasma renin levels and low serum aldosterone are also common findings (22).

In the present case, the patient was administered oral prednisone instead of hydrocortisone during hospitalization. Hydrocortisone should have been administered as the first-line therapy for the patient. However, in the present case, it was not possible to do so because hydrocortisone was unavailable in our hospital at the time, and therefore prednisone was used as a substitute. In limited-resource settings, sometimes the required medications are unavailable, necessitating flexibility in therapeutic management based on the patient's clinical condition. We decided to administer oral prednisone with the assumption that it is equivalent to hydrocortisone and would yield the same outcome for the patient.

To avoid adrenal crises, patients with adrenal insufficiency often require lifelong glucocorticoid replacement therapy and stress management (23). In this case, hydrocortisone was discontinued due to the patient’s good clinical condition and normal morning plasma cortisol levels. After a 1-year follow-up, the patient remained asymptomatic with normal cortisol levels, marking a noteworthy complete recovery, no previous studies have reported the same outcome as the present case. We hypothesized several reasons for this unique outcome: (i) The patient, at 38 years, was relatively young compared to previous cases, suggesting that an adequate immune system may play a role; (ii) despite a one-month delay in diagnosis and treatment, the absence of skin hyperpigmentation suggested an acute presentation, potentially contributing to the favorable outcome; and (iii) The absence of comorbidities such as diabetes mellitus, hypertension, or human immunodeficiency virus infection was documented in this case, potentially positively impacting the patient’s outcome.

On April 4, 2023, a search for published studies was conducted on PubMed and Google Scholar using a combination of keywords (‘Addison disease’ OR ‘Primary adrenal insufficiency’ OR ‘adrenal tuberculosis’) NOT Autoimmune AND Tuberculosis, with a title-only filter. The inclusion criteria were patients diagnosed with primary adrenal insufficiency caused by adrenal TB and studies published between 2000 and 2023. Studies published in languages other than English and those that were not randomized controlled trials, observational studies, or case reports were excluded. A total of 71 articles were identified from both databases and after removing duplicates and applying the eligibility criteria, 12 articles (10 case reports and two observational studies) were included in the review (see Table 1).

**Table 1:** Summary of clinical manifestations of primary adrenal insufficiency caused by adrenal TB infection.

Author	Research design	Sign and symptoms	Physical examination	Laboratory findings	Imaging test (abdominal)	Additional advanced examination
Msirdi *et al.* 2023 (9)	Case report	Asthenia, dizziness, chronic cough, and skin hyperpigmentation	Slightly dehydrated, bradycardia, hypotension	Mild hyponatremia, hyperkalemia, hypoglycemia, low cortisol level, high adrenocorticotropic level	CT scan: bilateral adrenal enlargement and calcifications	ECG: complete AV block sputum test: TB positive
Tran *et al.* 2021 (7)	Case report	Nausea, vomiting, diarrhea, abdominal pain, weakness, anorexia, weight loss, and skin hyperpigmentation	Hypotension and tachycardia	Low morning plasma cortisol, high plasma adrenocorticotropic hormone, high plasma renin concentration, hypoglycemia, mild hyponatremia	CT scan: bilateral adrenal masses	Adrenal biopsy: left adrenal gland granulomatous lesions with central caseous necrosis surrounded by lymphocytes and Langhans-type giant cells which were consistent with TB inflammation
Yang *et al.* 2021 (10)	Case report	Cardiac arrest, nausea, vomiting, loss of consciousness, fatigue, loss of appetite, weight loss	Hypotension	Low morning plasma cortisol, high plasma adrenocorticotropic hormone, high plasma renin concentration, hyponatremia, hyperkalemia	CT scan: multiple TB lesions throughout the body	TB-related test: a positive Mycobacterium TB conformity group DNA test, a positive rifampicin resistance gene test, and a positive TSPOT.TB test
Yu *et al.* 2020 (11)	Case report	Skin hyperpigmentation	Hypotension	Low morning plasma cortisol, high plasma adrenocorticotropic hormone	CT scan: bilateral adrenal hyperplasia	TSPOT.TB test: positive
Koh 2018 (12)	Case report	Skin hyperpigmentation, weakness, fever	Palpable mass on both sides of the neck, axilla, and inguinal areas	Low morning plasma cortisol, high plasma adrenocorticotropic hormone	CT scan: mass-like enlarged bilateral adrenal mass. rim enhancement, and focal cystic lesion	N/A
					^18^F-FDG PET CT: increased FDG uptake in both adrenal glands	
Soedarso *et al.* 2018 (13)	Case report	Depression, nausea, fatigue, weight loss, decreased appetite, abdominal pain, joint pain, skin hyperpigmentation	Normal	Low cortisol level	MRI: bilateral adrenal hypertrophy	Mantoux test: negative
						Gamma interferon (IFN-γ) release assay: negative
Sharma *et al.* 2016 (14)	Case report	Fever, fatigue, and loss of weight and appetite	Hypotension	Low albumin serum level, low cortisol level, hyponatremia, hyperkalemia	CT scan contrast-enhanced: bilateral adrenal enlargement	EUS-guided FNA: numerous acid-fast bacilli against a necrotic background
					PET-CT: uptake only in both adrenal glands	
					EUS: hypoechoic, left adrenal mass, with a definite outline	
Borgo *et al.* 2010 (15)	Case report	Fever, asthenia general malaise, fatigue, and night sweats	Mild cellulitis in his left elbow with moderate pain	Mild leukocytosis, abnormal values for the erythrocyte sedimentation rate, and C reactive protein level	CT scan: mass-like enlargement and rim enhancement without calcification	Mantoux test: negative
					MRI: heterogeneous enhancement and colliquative areas without calcification	Gamma interferon (IFN-γ) release assay for mycobacterial-specific proteins: positive
					US: enlarged bilateral glands with disomogeneous patterns and hypoechogenic areas inside	
Li *et al.* 2008 (16)	Case report	Fatigue, anorexia, nausea, vomiting, weight loss, skin hyperpigmentation	N/A	High serum cortisol levels and high adrenocorticotropic hormone	CT scan: mass-like enlargement of bilateral adrenal gland	N/A
					US: solid masses bilateral adrenal gland	
					MRI: irregular low signal intensity in the left adrenal mass on T2WI	
					^18^F-FDG PET CT: bilateral adrenal masses with increased FDG uptake	
Zhang *et al.* 2008 (17)	Observational study	–	–	–	MRI: bilateral involvement, T2 hypo- or isointense signal of central zone, and peripheral rim enhancement	–
Guo *et al.* 2007 (18)	Observational study	–	–	–	CT scan: bilateral enlargement, mass-like enlargement, peripheral rim, and calcification	–
Liatsikos *et al.* 2006 (19)	Case report	Weakness, lumbar pain, fever, weight loss, anorexia	Normal	Hyponatremia, hypoglycemia, low cortisol serum level	US: bilateral solid, hypoechogenic, and lobulated masses	Mantoux test: positive
					CT scan: bilateral adrenal enlargement	CT-guided FNA biopsy: caseous necrosis and granulomatous infection typical of TB
					CT scan contrast-enhanced: peripheral hyperattenuating rim with central, hypodense areas suggestive of necrotic lesions	

^18^F-FDG18F 2-fluoro-2-deoxy-d-glucose; AV, atrioventricular; EUS, endoscopic ultrasound; FNA, fine-needle aspiration;; TB, tuberculosis; US, ultrasonography.

By reviewing various studies, in limited resources setting, attending physicians can rely on a patient’s specific manifestation, such as skin hyperpigmentation, nausea, vomiting, abdominal pain, fatigue, weakness, decreased appetite, weight loss, and depressive symptoms, to differentiate diagnoses. A metabolic imbalance or its associated symptoms may prompt laboratory analysis of cortisol levels. The majority of individuals with adrenal TB have hyponatremia, hyperkalemia, hypoglycemia, and hypotension. If TSPOT.TB or gamma interferon (IFN-γ) release assays are unavailable, the Mantoux test is a reliable method for confirming TB infection. In cases where laboratory abnormalities are detected, abdominal grayscale ultrasonography, CT scans, and MRI examinations are recommended to confirm whether adrenal insufficiency is due to TB or other adrenal malignant diseases. Abdominal grayscale ultrasonography can be particularly useful in limited resource settings, as it can reveal solid masses in the bilateral adrenal glands. CT scans may show bilateral adrenal enlargement and calcifications, while MRI scans may reveal bilateral involvement, T2 hypointense or isointense signals in the central zone, and peripheral rim enhancement.

## Declaration of interest

The authors declare that there is no conflict of interest that could be perceived as prejudicing the impartiality of the case study reported.

## Funding

This study did not receive any specific grant from any funding agency in the public, commercial, or not-for-profit sector.

## Informed consent

Written informed consent for publication of their clinical details and/or clinical images was obtained from the patient.

## Patient’s perspective

I was frustrated back then because I had visited two hospitals, and the doctors kept referring me to another hospital. I really wanted to regain my normal health and lifestyle. As you know, being sick also had an impact on my family. I felt sorry for them. I’m incredibly grateful that after being hospitalized at Dr. Zainoel Abidin Hospital, I no longer experience the sickness, and I was able to return to my normal activities after 6 months.

## Author contribution statement

HZ: conceptualization, validation, supervision, writing – review and editing, funding acquisition; RR: writing – original draft preparation, data collection; POZ: writing – original draft preparation, review, and editing; KWS, AED, and SF: validation, supervision, writing – review and editing; all authors approved the submitted version of the manuscript.
